# LncRNA KCNQ1OT1 sponges miR-34c-5p to promote osteosarcoma growth via ALDOA enhanced aerobic glycolysis

**DOI:** 10.1038/s41419-020-2485-1

**Published:** 2020-04-24

**Authors:** Yifei Shen, Jingwen Xu, Xiaohui Pan, Yunkun Zhang, Yiping Weng, Dong Zhou, Shisheng He

**Affiliations:** 1 Department of Orthopedics, Shanghai Tenth People’s Hospital, School of Medicine, Shanghai Tongji University, 200120 Shanghai, China; 2 Department of Nutrition, Changzhou No.2 People’s Hospital, Nanjing Medical University, 213003 Changzhou, Jiangsu China; 3 Department of Orthopedics, Changzhou No.2 People’s Hospital, Nanjing Medical University, 213003 Changzhou, Jiangsu China; 4 Department of Orthopedics, Wuqia People’s Hospital, 845450 Xinjiang, China

**Keywords:** Bone cancer, Cell growth

## Abstract

Metabolic switch from oxidative phosphorylation to aerobic glycolysis, which is also called the Warburg effect, is a hallmark of osteosarcoma (OS) and leads to the enhancement of cell chemoresistance, growth, metastasis, and invasion. Emerging evidence indicates that long non-coding RNA (lncRNA) plays a crucial role in the Warburg effect of cancer cells. Here, we report that lncRNA KCNQ1OT1 was upregulated in OS. Meanwhile, functional experiments demonstrated that the KCNQ1OT1 facilitated proliferation and suppressed apoptosis of OS cells. In addition, KCNQ1OT1 contributed to the Warburg effect by stimulating aldolase A (ALDOA) expression. Furthermore, using bioinformatics analysis, luciferase reporter, RNA immunoprecipitation, and RNA pull-down assay, we identified that KCNQ1OT1 functions as a competing endogenous RNA (ceRNA) by sponging miR-34c-5p, which inhibited ALDOA expression by directly targeting its 3ʹUTR. Taken together, these data identified a key role of KCNQ1OT1 in glucose metabolism reprogramming of OS. Targeting the KCNQ1OT1/miR-34c-5p/ALDOA axis may be a potential therapeutic target in OS treatment.

## Introduction

Osteosarcoma (OS) is one of the most aggressive and common primary malignant bone tumors, and it occurs most frequently in children and adolescents^1^. Due to advances in various treatment strategies such as surgery, chemotherapy, and radiotherapy, the five-year survival rate of OS in non-metastatic OS patients has reached 60%^2^. However, the overall survival rate has not markedly increased over the last decade because the molecular mechanisms underlying the pathogenesis and progression of OS are poorly understood^3^. Thus, it is of great importance to explore the molecular mechanisms related with the OS progression and pathogenesis and to identify more effective therapeutic targets.

Aerobic glycolysis is a general feature of energy metabolism in cancer cells, and it is also termed the Warburg effect^4–6^. Although aerobic glycolysis is less efficient in the generation of adenosine triphosphate (ATP), it increases proliferation, inhibits apoptosis, and generates signaling metabolites to enhance cancer cell survival under molecularly stressful conditions^7,8^. Fructose-bisphosphate aldolase A (ALDOA), a glycolytic enzyme, plays an important role in glycolysis and gluconeogenesis^9^. Notably, ALDOA is highly expressed and acts as an oncogene in many types of cancers including lung squamous cell carcinomas, colorectal cancer, hepatocellular carcinomas, and oral squamous cell carcinomas^10–13^. Previous studies have reported that ALDOA is upregulated in OS and is a negative survival marker of these patients^14,15^. However, the role of ALDOA in OS progression and its underlying molecular mechanisms remain unclear.

Long non-coding RNA (lncRNA) is a subtype of RNA longer than 200 nucleotides that lacks protein-coding functions^16^. The lncRNA plays a crucial role in regulating gene expression at different stages including chromatin remodeling, transcription, and post-transcriptional regulation^17,18^. Notably, lncRNA also functions as a competing endogenous RNA (ceRNA) that sponges microRNAs (miRNAs) that target messenger RNA (mRNA) expression^19,20^. Recent reports have shown that different lncRNA transcripts play important roles in initiation, progression, and development of multiple tumors as either oncogenes or tumor suppressor genes^21–23^. KCNQ1 opposite strand/antisense transcript 1 (KCNQ1OT1) is a type of long chromatin-interacting lncRNA that has been widely reported to be a cancer promoter in various types of tumors such as non-small cell lung carcinoma, colorectal cancer, tongue cancer, and breast cancer^24–27^.

In this study, we demonstrated that KCNQ1OT1 was upregulated in OS and promoted tumor growth by contributing to aerobic glycolysis. Mechanistically, KCNQ1OT1 competes with a key glycolytic coding mRNA, ALDOA, for miR-34c-5p and relieves the inhibitory effect of miR-34c-5p on ALDOA, thereby leading to increased ALDOA expression and aerobic glycolysis. Thus, the KCNQ1OT1-miR-34c-5p/ALDOA axis may provide a new therapeutic target for OS treatment.

## Results

### LncRNA KCNQ1OT1 was upregulated in OS and promoted the growth of the OS cells

**Fig. 1:**
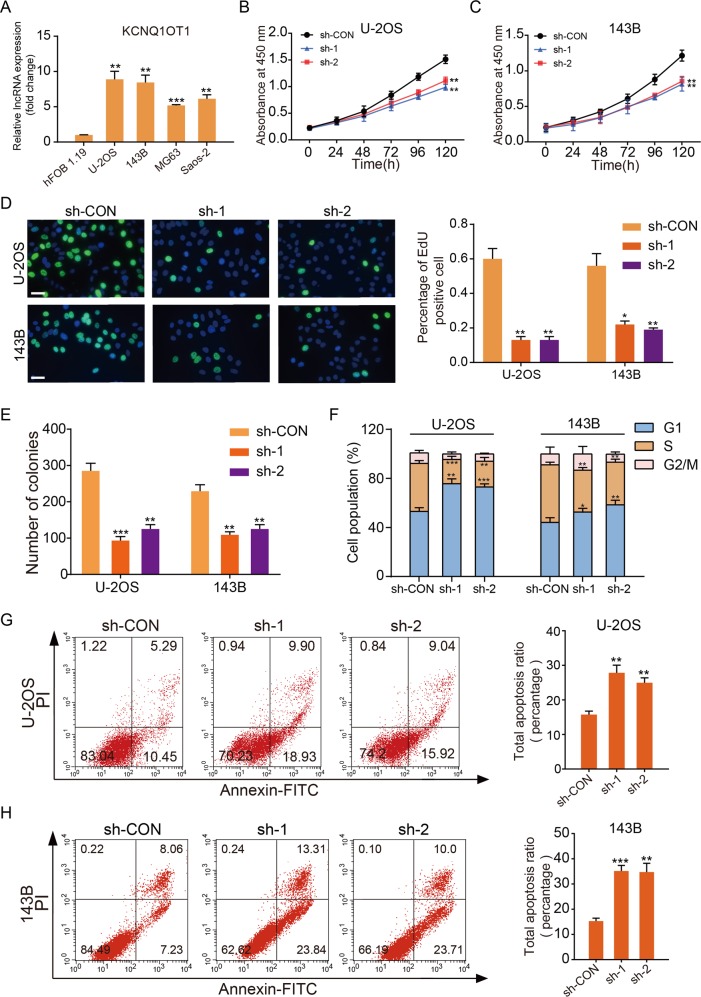
KCNQ1OT1 promoted proliferation and inhibited the apoptosis of OS cells in vitro. **a** The expression patterns of KCNQ1OT1 in hFOB1.19, a normal osteoblast cell line, and OS cell lines (U-2OS, 143B, MG63, and Saos-2) by qRT-PCR. Values are means ± SD, **p < 0.01, ***p < 0.001 (Student’s *t*-test). **b** and **c** Knockdown of KCNQ1OT1 suppressed proliferation capability of U-2OS and 143B cells using the CCK-8 assay. Values are means ± SD, **p < 0.01 (Student’s *t*-test). **d** KCNQ1OT1 shRNA decreased the percentage of EdU-positive OS cells. Representative photographs of the EdU incorporation assay were shown in the left panel, Scale bars = 100 μm. Values are means ± SD, *p < 0.05, **p < 0.01. **e** Knockdown of KCNQ1OT1 suppressed the proliferation of OS cells (U-2OS and 143B) using the colony formation assay. Values are means ± SD, **p < 0.01, ***p < 0.001 (Student’s *t*-test). **f** The cell cycle was determined in the U-2OS cells and 143B cells by flow cytometry after transfected with the KCNQ1OT1 sh-RNA. The diagrams quantified cell fractions in the G_1_, S, and G_2_/M fractions. Values are means ± SD, *p < 0.05, **p < 0.01, ***p < 0.001 (Student’s *t*-test). **g** and **h** The knockdown of KCNQ1OT1 significantly induces apoptosis of U-2OS and 143B cells. Values are means ± SD, **p < 0.01, ***p < 0.001 (Student’s *t*-test).

In order to explore the role of KCNQ1OT1 in OS progression, we first determined the expression patterns of KCNQ1OT1 via comparing the expression levels of KCNQ1OT1 in four OS cell lines (U-2OS, 143B, MG63, and Saos-2), with that of a normal human osteoblast cell line (hFOB1.19) via real-time polymerase chain reaction (qRT-PCR). As displayed in Fig. 1a, all of the OS cell lines exhibited significantly higher expression of KCNQ1OT1 than the control human osteoblast cell line, especially in the U-2OS and 143B cells. Further exploration of the biological function of KCNQ1OT1 in the progression of OS, involved two short hairpin RNA (sh-1, sh-2) against KCNQ1OT1 and the non-targeting shRNA (sh-CON) were designed and stable KCNQ1OT1 knockdown U-2OS and 143B cell lines were established. The knockdown efficiency was evaluated by qRT-PCR (Supplementary Fig. 1a). The cell counting kit-8 (CCK-8) assay (Fig. 1b, c), EdU assay (Fig. 1d), colony formation assay (Fig. 1e and Supplementary Fig. 1b), and cell cycle assay (Fig. 1f and Supplementary Fig. 1c, d) showed that knockdown of KCNQ1OT1 partly inhibited the proliferation of U-2OS and 143B cells. Next, we evaluated the effects of KCNQ1OT1 knockdown on cell apoptosis. As depicted in Fig. 1g, h, compared with the control cells, downregulation of KCNQ1OT1 significantly induced the apoptosis of the OS cells.

**Fig. 2:**
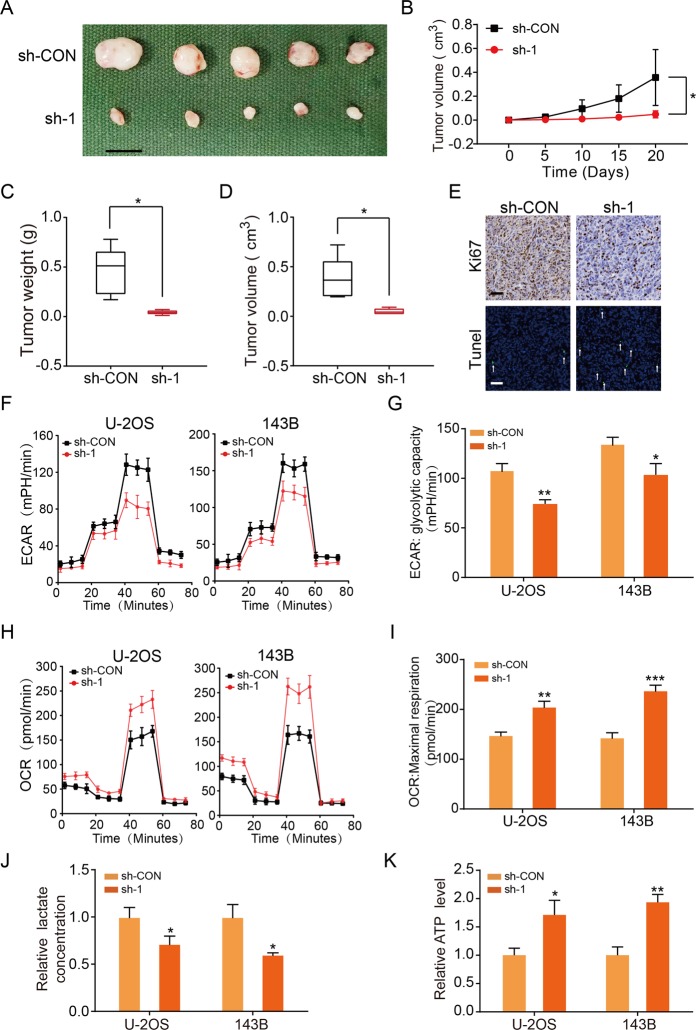
KCNQ1OT1 facilitates OS proliferation in vivo and promoted aerobic glycolysis in OS cells. **a** Morphologic characteristics of xenograft tumors from U-2OS/sh-Control group and U-2OS/sh-KCNQ1OT1 group (*n* = 5). Scale bars = 1 cm. **b** The tumor volumes were measured with calipers every 5 days. Values are means ± SD, *p < 0.05 (Student’s *t*-test). **c** Tumor weights at 20 days were measured in each group. The median, upper, and lower quartiles were plotted, and the whiskers that extend from each box indicate the range of values that were outside of the intra-quartile range. *n* = 5, *p < 0.05 (Student’s *t*-test). **d** Tumor volumes at 20 days were measured in each group. The median, upper, and lower quartiles were plotted, and the whiskers that extend from each box indicate the range values that were outside of the intra-quartile range. *n* = 5, *p < 0.05 (Student’s *t*-test). **e** Representative images of Ki67 and TUNEL staining in the xenograft tumors from the sh-KCNQ1OT1 and sh-Control mice. A TUNEL positive cell is indicated (arrow). **f** Extracellular acidification rate (ECAR) of U-2OS or 143B cells in sh-Control and the sh-KCNQ1OT1 group was detected via a Seahorse Bioscience XFp analyzer. Glc: glucose, Oligo: oligomycin, 2-DG: 2-deoxy-d-glucose. **g** Quantification of the glycolytic capacity from the Fig. 2f, *p < 0.05, **p < 0.01 (Student’s *t*-test). **h** O_2_ consumption rate (OCR) of U-2OS or 143B cells in sh-Control and sh-KCNQ1OT1 group was detected using a Seahorse Bioscience XFp analyzer. O: Oligomycin, F: FCCP, A&R: antimycin A/rotenone. **i** Quantification of maximal respiration from the Fig. 2h, **p < 0.01, ***p < 0.001 (Student’s *t*-test). **j** Lactate production was determined in the U-2OS and 143B cells stable knockdown KCNQ1OT1. Values are means ± SD, *p < 0.05 (Student’s *t*-test). **k** ATP level was determined in U-2OS and 143B cells stable knockdown KCNQ1OT1. Values are means ± SD, *p < 0.05, **p < 0.01 (Student’s *t*-test).

Furthermore, to determine whether KCNQ1OT1 promoted growth of the OS cells in vivo, we utilized xenograft experiments by subcutaneously injecting stable KCNQ1OT1-knockdown or control U-2OS cells into nude mice. We found that the stable KCNQ1OT1-knockdown group exhibited significantly decreased the xenografted tumor growth and a decreased tumor burden compared to the control group (Fig. 2a–d). Immunohistochemical staining and terminal deoxynucleotidyl transferase dUTP nick-end labeling (TUNEL) assay revealed declining expression of Ki67 and a rising rate of apoptosis in the xenografted tumors of KCNQ1OT1-knockdown group (Fig. 2e). To further explore the mechanism of regulation of KCNQ1OT1 on cell proliferation and apoptosis, we detected several apoptosis-related and cell cycle-related molecules by western blotting. The results showed that knockdown of KCNQ1OT1 effectively promoted the activated levels of cleaved Caspase-3, cleaved Caspase-7 and cleaved Caspase-9 and inhibit the expression of cyclin-dependent kinase 4 (CDK4) and Cyclin D1 (Supplementary Fig. 2a). These data further indicate that KCNQ1OT1 was upregulated in OS cell lines and promoted cell survival through inhibiting cell apoptosis and facilitating proliferation of OS cells both in vitro and in vivo.

### KCNQ1OT1 contributed to the Warburg effect in OS

It has been widely accepted that metabolic shift from oxidative phosphorylation to aerobic glycolysis, also called the Warburg effect, contributes to the promotion of growth and apoptosis avoidance in tumors^28^. To assess whether KCNQ1OT1 contributes to bioenergetic changes in OS, we used a metabolic flux analyzer to measure extracellular acidification rate (ECAR) and the oxygen consumption rate (OCR) in KCNQ1OT-knockdown and control OS cells. Silencing KCNQ1OT1 significantly suppressed the ECAR in U-2OS and 143B cells (Fig. 2f, g) but enhanced OCR in these cells (Fig. 2h, i). Furthermore, knockdown of KCNQ1OT1 in OS cells also promoted the level of ATP produced by oxidative phosphorylation and decreased the formation of lactate produced by aerobic glycolysis (Fig. 2j, k). Collectively, these results showed that KCNQ1OT1 inhibited oxidative phosphorylation and facilitated aerobic glycolysis in OS.

### KCNQ1OT1 promoted OS growth via ALDOA-mediated glycolysis

**Fig. 3:**
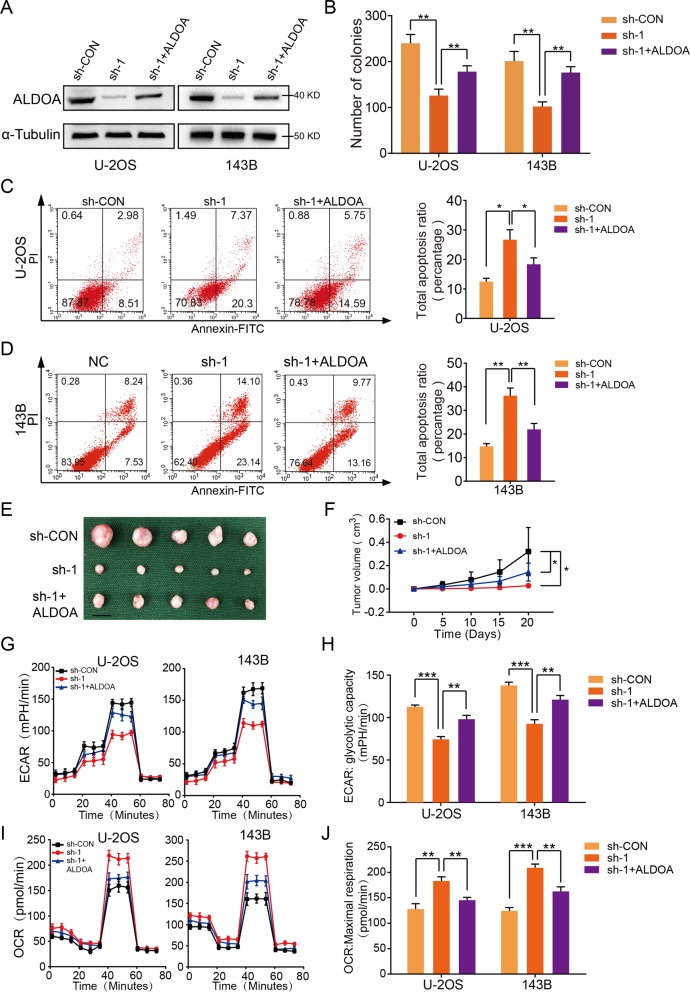
KCNQ1OT1 enhanced OS growth by modulating ALDOA expression. **a** Overexpression efficacy of ALDOA in sh-KCNQ1OT1 OS cells (U-2OS and 143B) was detected by western blotting. **b** Overexpression of ALDOA partly reversed the suppressed effects of KCNQ1OT1-knockdown on the colony formation capability of U-2OS and 143B cells, values are means ± SD, **p < 0.01 (Student’s *t*-test). **c** and **d** Overexpression of ALDOA partly reversed the induced effect of KCNQ1OT1-knockdown on the apoptosis of OS cells (U-2OS and 143B). Values are means ± SD, *p < 0.05, **p < 0.01 (Student’s *t*-test). **e** Morphologic characteristics of the xenograft tumors from U-2OS/sh-Control group, U-2OS/sh-KCNQ1OT1 group and U-2OS/sh-KCNQ1OT1 + ALDOA group (*n* = 5). Scale bars = 1 cm. **f** Overexpression of ALDOA partly rescued the inhibitory effects of KCNQ1OT1-knockdown on the growth rate of U-2OS cells in vivo. The volumes of tumors were measured every 5 days; values are means ± SD, *p < 0.05 (Student’s *t*-test). **g** The ECAR in OS cells (MNNG-HOS and U-2OS) in different groups (sh-Control, sh-KCNQ1OT1, and sh-KCNQ1OT1 + ov-ALDOA) were determined. Values are means ± SD. **h** Quantification of the glycolytic capacity from Fig. 3g, **p < 0.01, ***p < 0.001 (Student’s *t*-test). **i** The OCR in OS cells (U-2OS and 143B) in different groups (sh-Control, sh-KCNQ1OT1, and sh-KCNQ1OT1 + ov-ALDOA) were determined. Values are means ± SD. **j** Quantification of maximal respiration from the Fig. 3i, **p < 0.01, ***p < 0.001 (Student’s *t*-test).

To investigate how KCNQ1OT1 regulated glucose metabolism in OS, a panel of genes involved in the glycolysis pathway and tricarboxylic acid (or Krebs) cycle were measured by qRT-PCR in U-2OS and 143B cells with altered KCNQ1OT1 expression. qRT–PCR analysis showed that knockdown of KCNQ1OT1 significantly suppressed mRNA expression of ALDOA, a key glycolytic gene, in U-2OS and 143B cells (Supplementary Fig. 3a, b). We also compared the expression level of ALDOA in OS cell lines (U-2OS, 143B, and MG63), with that in hFOB1.19. As shown in Supplementary Fig. 3c, all these OS cell lines showed significantly higher expression of ALDOA than the hFOB1.19. Next, we determined whether KCNQ1OT1 participates in OS growth by regulating ALDOA-mediated glycolysis. First, we overexpressed ALDOA in wild-type (WT) and KCNQ1OT1 knockdown OS cell lines and confirmed overexpression efficiency by western blot (Fig. 3a). As shown in Fig. 3b–d and Supplementary Fig. 3d, overexpression of ALDOA partly reversed the inhibited effects of KCNQ1OT1 knockdown on the growth-promoting properties of OS cells. The results of in vivo xenograft experiments showed that anti-tumorigenic effects on KCNQ1OT1-knockdown group were partly reversed by ALDOA overexpression (Fig. 3e, f). In addition, ALDOA overexpression also partly restored the Warburg effect in U-2OS and 143B cells (Fig. 3g–j). Kaplan–Meier analysis from the R2 database was used to determine the prognostic significance of ALDOA in OS patients. As shown in Supplementary Fig. 3e, high ALDOA expression contributed to a lower overall survival rate in patients with OS. Collectively, these results validated that KCNQ1OT1 influences ALDOA-mediated glycolysis and then promotes OS growth.

### miR-34c-5p directly targeted ALDOA in OS cells

**Fig. 4:**
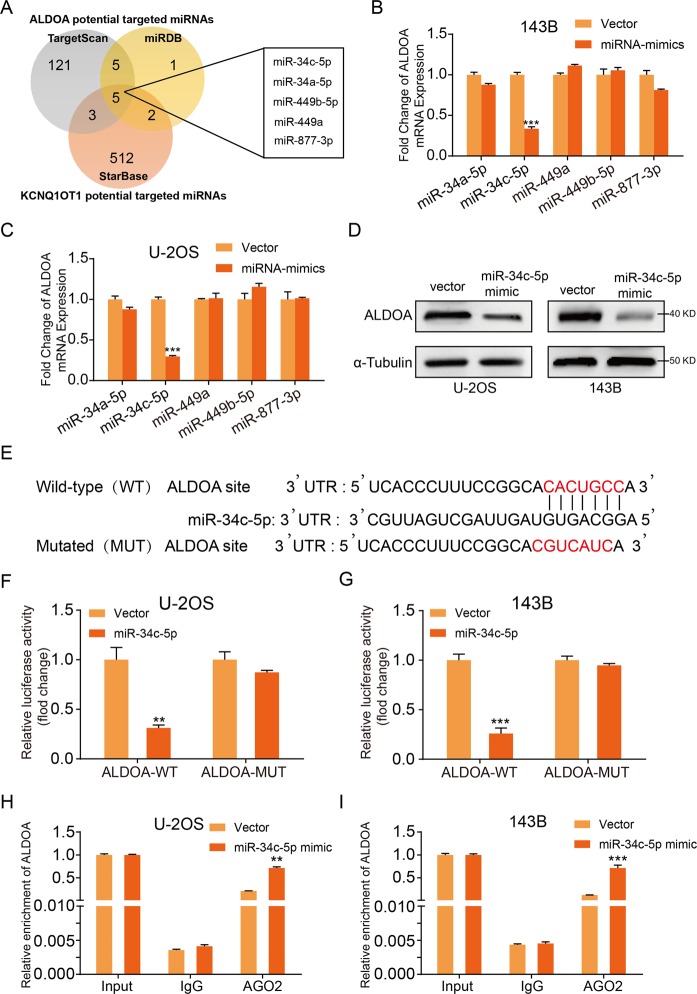
ALDOA was a direct target of miR-34c-5p. **a** Venn diagram showing the predicted target genes of ALDOA and KCNQ1OT1 from databases (miRDB, TargetScan, and StarBase). **b** and **c** The mRNA expression patterns of the ALDOA in predicted target miRNA mimics the treated or Control U-2OS and 143B cells, ***p < 0.001 (Student’s *t*-test). **d** Western blot showed the ALDOA expression in the U-2OS and 143B cells transfected with miR-34c-5p mimics or negative control. **e** The wild-type and the mutated sequences of the ALDOA mRNA 3’-UTR (mutation site: red). **f** and **g** The luciferase activity of the OS cells (U-2OS and 143B) in luciferase reporter plasmid containing wild-type ALDOA 3’-UTR (ALDOA-WT) and mutant ALDOA 3’-UTR (ALDOA-MUT) co-transfected with miR-34c-5p mimics or negative control was assessed, **p < 0.01, ***p < 0.001 (Student’s *t*-test). **h** and **i** RIP assays using antibodies against AGO2 or IgG were performed in cellular lysates from U-2OS and 143B cells. qRT-PCR showed the relative enrichment of ALDOA in cells transfected with miR-34c-5p or NC mimics, **p < 0.01, ***p < 0.001 (Student’s *t*-test).

It has been reported that some lncRNA sponges microRNAs to regulate the target gene expression. We found five potential microRNAs that target ALDOA and are regulated by KCNQ1OT1 by overlap in three commonly used prediction algorithms TargetScan, miRDB and Starbase (Fig. 4a). We therefore overexpressed these above five miRNAs in U-2OS and 143B cells by transfecting miRNA mimics. Overexpression efficiency was confirmed by qRT-PCR (Supplementary Fig. 4a, b). Overexpression of miR-34c-5p significantly suppressed mRNA and protein expression of ALDOA in U-2OS and 143B cells (Fig. 4b–d). Furthermore, hFOB1.19 showed significantly higher expression of miR-34c-5p than the OS cell lines (Supplementary Fig. 4c). The potential target site of miR‑34c-5p on ALDOA is shown in Fig. 4e. Dual luciferase reporter assays were used to further confirm that miR-34c-5p directly targets ALDOA. These results showed that the miR-34c-5p mimic led to a significant reduction in the luciferase activity of the WT ALDOA 3′UTR (ALDOA-WT) reporter, but not in the Mut 3′UTR of ALDOA reporter U-2OS and 143B cells (Fig. 4f–g). Furthermore, RNA immunoprecipitation (RIP) assay revealed a higher enrichment level of ALDOA in the Argonaute 2 (Ago2) group after overexpression of miR-34c-5p (Fig. 4h–i). Taken together, these data showed that miR-34c-5p inhibited ALDOA expression by directly targeting its 3ʹUTR.

### KCNQ1OT1 functioned as a ceRNA by sponging miR-34c-5p

**Fig. 5:**
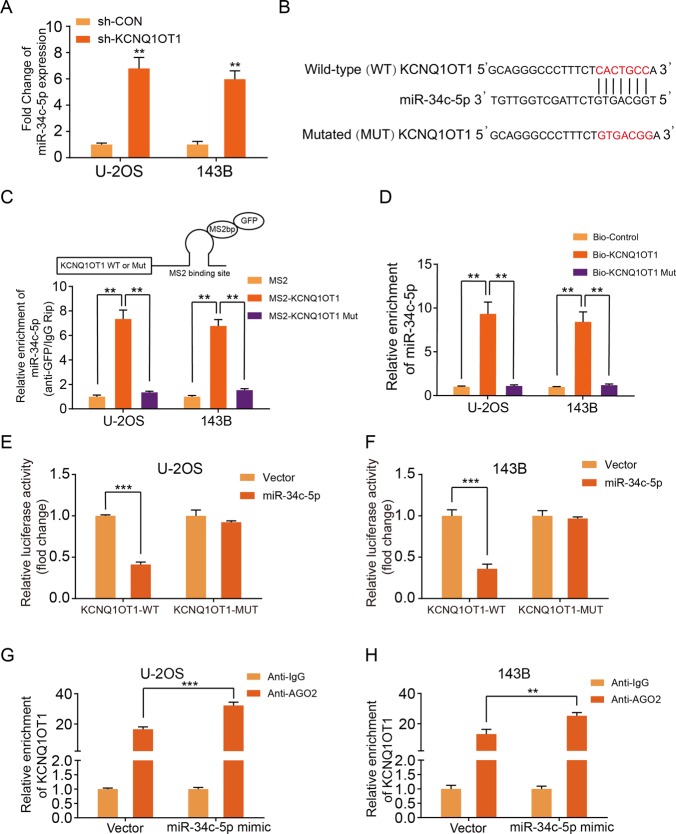
KCNQ1OT1 bound to miR-34c-5p and decreased expression in the OS cells. **a** The expression of miR-34c-5p was upregulated in U-2OS and143B transfected with KCNQ1OT1 shRNA or the control shRNA by RT-qPCR, **p < 0.01, ***p < 0.001 (Student’s *t*-test). **b** The wild-type and the mutated sequences of the KCNQ1OT1 mRNA 3’-UTR (mutation site: red). **c** Top panel shows a schematic image of a construction containing KCNQ1OT1 wild type combined with MS2 binding sequence. MS2-RIP followed by miR-34c-5p qRT-PCR to measure miR-34c-5p endogenously associated with KCNQ1OT1, **p < 0.01 (Student’s *t*-test). **d** U-2OS and 143B cells lysate were incubated with biotin-labeled KCNQ1OT1, qRT-PCR measured miR-34c-5p expression in the products of pulldown by biotin, **p < 0.01 (Student’s *t*-test). **e** and **f** The luciferase activity of the OS cells (U-2OS and 143B) in luciferase reporter plasmid containing wild-type KCNQ1OT1 3’-UTR (KCNQ1OT1-WT) and mutant KCNQ1OT1 3’-UTR (KCNQ1OT1-MUT) co-transfected with miR-34c-5p mimics or negative control was assessed, ***p < 0.001 (Student’s *t*-test). **g** and **h** AGO2-RIP followed by qPCR to evaluate KCNQ1OT1 level after miR-34c-5p overexpression, **p < 0.01, ***p < 0.001 (Student’s *t*-test).

To further investigate whether KCNQ1OT1 acted as a sponge to miR-34c-5p in OS cells, we measured the miR-34c-5p expression in KCNQ1OT1-knockdown and control U-2OS and 143B cells. Silencing of KCNQ1OT1 significantly increased the expression level of miR-34c-5p in OS cells (Fig. 5a). Furthermore, bioinformatics analysis revealed that ALDOA contains a putative binding site of miR-34c-5p (Fig. 5b). Meanwhile, we constructed plasmids containing either WT or mutated type (MUT) miR-34c-5p binding site of the KCNQ1OT1 transcript combined with an MS2 binding site and co-transfected these into U-2OS or 143B cell lines with a construct containing MS2-binding protein (MS2bp) and GFP. An MS2-based RIP assay was performed, and as shown in Fig. 5c, miR-34c-5p was significantly enriched in RNAs retrieved from the WT MS2bs-KCNQ1OT1 group compared with the MUT MS2bs-KCNQ1OT1 group. RNA pull-down assay further revealed that miR-34c-5p bound biotin-labeled WT KCNQ1OT1 but not MUT KCNQ1OT1 (Fig. 5d). Subsequently, a luciferase reporter construct containing KCNQ1OT1 (WT or MUT miR-34c-5p binding site) was generated and co-transfected into U-2OS cells and 143B cells with miR-34c-5p mimics. The results of luciferase assay showed that upregulation of miR-34c-5p enhanced the luciferase activity of WT KCNQ1OT1 but not MUT KCNQ1OT1 (Fig. 5e, f). Ago2 is the essential component of the RNA-induced silencing complex (RISC)^29^. To determine whether KCNQ1OT1 binds miR-34c-5p in an Ago2-dependent manner, we performed anti-Ago2 RIP in U-2OS and 143B cells transiently overexpressing miR-34c-5p. Endogenous KCNQ1OT1 enrichment was increased after overexpression of miR-34c-5p in OS cells (Fig. 5h, i). Taken together, these results indicated that KCNQ1OT1 sponges miR-34c-5p and may function as a ceRNA in OS.

### MiR-34c-5p inhibition reversed the inhibitory effect of OS cells induced by KCNQ1OT1 depletion

**Fig. 6:**
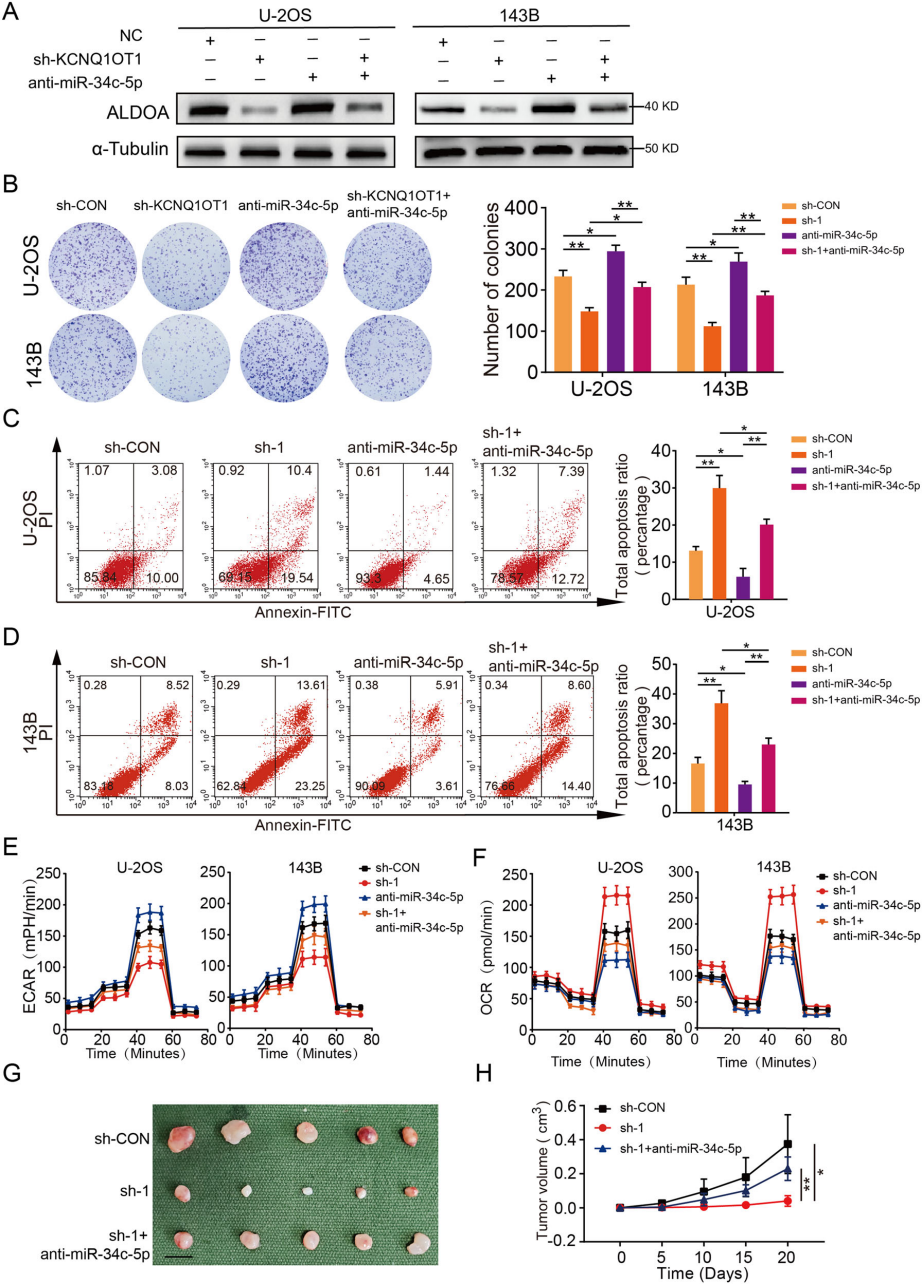
MiR-34c-5p inhibition partly rescued the KCNQ1OT1 knockdown effect in OS cells. **a** Western blot showed the ALDOA expression in U-2OS and 143B cells transfected with miR-34c-5pinhibitor, sh-KCNQ1OT1, or negative control. **b** miR-34c-5-knockdown partly reversed the inhibitory effects of KCNQ1OT1-knockdown on the colony formation properties of U-2OS and 143B cells and silenced miR-34c-5 in wide type OS cells (U-2OS and 143B) also promoted their proliferation, values are means ± SD, *p < 0.05, **p < 0.01 (Student’s *t*-test). **c** and **d** miR-34c-5-knockdown partly reversed the induce effect of KCNQ1OT1-knockdown on the apoptosis of OS cells (U-2OS and 143B). Knockdown of miR-34c-5 inhibited apoptosis of wide OS cells. Values are means ± SD, *p < 0.05, **p < 0.01 (Student’s *t*-test). **e** Altered levels of ECAR in the OS cells (U-2OS or 143B) in different groups (sh-Control, sh-KCNQ1OT1, sh-KCNQ1OT1 + anti-miR-34c-5p and anti-miR-34c-5p). Values are means ± SD. **f** Altered levels of OCR in the OS cells (U-2OS or 143B) in different groups (sh-Control, sh-KCNQ1OT1, sh-KCNQ1OT1 + anti-miR-34c-5p and anti-miR-34c-5p). Values are means ± SD. **g** Morphologic characteristics of xenograft tumors from U-2OS/sh-Control group, U-2OS/sh-KCNQ1OT1 group and U-2OS/sh-KCNQ1OT1 + anti-miR-34c-5p group (*n* = 5). Scale bars = 1 cm. **h** Anti-miR-34c-5p partly rescued the inhibitory effects of KCNQ1OT1-knockdown on the growth rate of U-2OS cells in vivo. The volumes of tumors were measured every 5 days; values are means ± SD, *p < 0.05, **p < 0.01 (Student’s *t*-test).

To explore whether KCNQ1OT1 exerted its function in OS cells through miR-34c-5p, we performed a rescue experiment by transfecting sh-KCNQ1OT1 plus miR-34c-5p inhibitors into U-2OS and 143B cells. The knockdown efficiency of miR-34c-5p inhibition was evaluated by RT-qPCR (Supplementary Fig. 5a). We found that downregulation of KCNQ1OT1 significantly inhibited protein expression of ALDOA, while the inhibition of miR-34c-5p partially rescued ALDOA expression in U-2OS and 143B cells (Fig. 6a). As shown in Fig. 6b–d, silence of miR-34c-5p reduced the inhibitory effect of KCNQ1OT1 knockdown on the growth of OS cells. Similarly, miR-34c-5p inhibition also partly restored the Warburg effect in U-2OS and 143B cells (Fig. 6e, f and Supplementary Fig. 5b, c). In an in vivo assay, a compromised tumorigenic potential in the KCNQ1OT1-knockdown group was partly offset via the silence of miR-34c-5p (Fig. 6g, h). Finally, the expression pattern of KCNQ1OT1/miR-34c-5p/ALDOA axis in human OS tissues and xenograft tumors were validated through fluorescence in situ hybridization (FISH) and immunofluorescence (IF) staining. A positive correlation between the expression pattern of KCNQ1OT1 and ALDOA, and a negative correlation between KCNQ1OT1 and miR-34c-5p, as well as miR-34c-5p and ALDOA were apparent (Supplementary Fig. 5d–g). Similarly, knockdown of KCNQ1OT1 also promoted the expression of miR-34c-5p and inhibited the expression of ALDOA in tumor tissues from a subcutaneous xenograft mouse model (Supplementary Fig. 5h). These results validated that KCNQ1OT1 promotes OS growth and the Warburg effect via serving as a ceRNA to sponge miR-34c-5p.

## Discussion

Accumulating evidence reports that dysregulated lncRNA is related to OS patient outcomes and affected the cancer progress. Yu et al.^30^ found that lncRNA taurine upregulated gene 1 (TUG1) promotes OS cell metastasis by mediating the miR-143-5p/HIF-1α pathway. Wang et al.^31^ revealed that lncRNA small nucleolar RNA host gene 16 (SNHG16) was highly expressed in OS and contributed to the growth of OS by promoting BCL9 expression via sponging miR-1301. Zhang et al.^32^ demonstrated that elevated long intergenic non-coding RNA 161 (LINC00161) reversed the cisplatin-resistant phenotype of OS cells by upregulating interferon-induced protein with tetratricopeptide repeats 2 (IFIT2). In this study, we discovered upregulated lncRNA KCNQ1OT1 in OS cell lines and tissues. In addition, KCNQ1OT1 promoted OS growth in vivo and also repressed apoptosis and stimulated cell proliferation in vitro. However, KCNQ1OT1 knockdown had little effect on the growth of the normal human osteoblast cell line hFOB1.19 (Supplementary Fig. 6a–c). Our findings revealed that KCNQ1OT1 was oncogenic and played a specific role in the occurrence and progression of OS.

LncRNA KCNQ1OT1 has been reported to be involved in cancer cell chemoresistance, growth, and invasion in a cell-type dependent manner^33^. In cholangiocarcinoma (CCA), KCNQ1OT1 was highly expressed and acted as a ceRNA to improve CCA proliferation and invasion by regulating the miR-140-5p/SOX4 axis^34^. Previous work demonstrated that KCNQ1OT1 inhibited the chemosensitivity to cisplatin (CDDP) through upregulation of KCNQ1OT1 via DNA methyltransferase (DNMT) in OS cell line MG-63^35^. In our study, we also showed that treatment of CDDP or sh-KCNQ1OT1 facilitated apoptosis of 143B and U-2OS cells, and the combination of CDDP and sh-KCNQ1OT1 enhanced this function (Supplementary Fig. 6d, e). More importantly, we elucidated the role of the KCNQ1OT1 in metabolic alterations in OS cells. We reported that silencing KCNQ1OT1 significantly decreased aerobic glycolysis and increased mitochondrial respiration in OS cells.

Increased aerobic glycolysis (the Warburg effect) facilitates cancer cell growth and survival, especially by providing more intermediates for many biosynthetic pathways and adapting to a hypoxic condition^36–39^. Therefore, targeting glycolysis has been demonstrated to be an effective method for controlling tumor growth and enhancing anticancer therapies. However, little is known about the role of lncRNA in glucose metabolism reprogramming in cancer cells. In this study, we report that KCNQ1OT1 is a novel promoter of the Warburg effect in OS by sponging miR-34c-5p, which directly targets the 3ʹUTR of ALDOA, a critical glycolytic enzyme.

ALDOA catalyzes the reversible conversion of fructose-1,6-bisphosphate to glyceraldehyde-3-phosphate (GAP) and dihydroxyacetone phosphate (DHAP)^40^. It is widely accepted that ALDOA is highly expressed and is correlated with poor survival outcomes in many kinds of harmful cancers^10–13^. We also found that ALDOA is upregulated in OS cells and tissues and related with worse overall survival rates. Our findings indicated that ALDOA contributed to the Warburg effect and enhanced growth of OS cells but not hFOB1.19 cells (Supplementary Fig. 6f–j).

In conclusion, our study identified KCNQ1OT1 as an oncogene that was highly expressed in OS. We suggest an important role for KCNQ1OT1 in glucose metabolism reprogramming via competitively binding miR-34c-5p to facilitate ALDOA expression in OS. These findings imply that KCNQ1OT1 may be a potential clinical therapeutic target for patients with OS.

## Materials and methods

### Cell culture

Two human osteosarcoma cell lines (MG63 and Saos-2) and human osteoblast hFOB1.19 cells were provided by the Cell Bank of the Chinese Academy of Sciences (Shanghai, China). The human osteosarcoma U-2OS and 143B cell lines were obtained from the American Type Culture Collection (ATCC, Manassas, VA, USA). Mycoplasma contamination testing was performed using Mycoplasma genus-specific PCR. Each cell line passed the DNA profiling (STR) test. All of the cell lines were cultured on the basis of ATCC protocols. MG63, Saos-2, U-2OS, and 143B were incubated in a 5% CO_2_ atmosphere at 37 °C. Moreover, the hFOB1.19 cells were cultured in a 5% CO_2_ atmosphere at 34.5 °C.

### Quantitative RT-PCR (qRT-PCR)

Total RNA from the cells was extracted and reverse transcribed as described before^41^. Real-time PCR analyses for miRNA or mRNA were performed on a 7500 Real-time PCR system (Applied biosystems) as previously described^42^. β-actin and U6 served as internal controls. All primers were listed in Supplementary Table 1.

### Cell transfection

The sequences of shRNAs targeting KCNQ1OT1 were sh-1, 5′-GCCAATAGCAACTGACTAA-3′ and sh-2, 5′-GCCACATCTAACACCTATA-3′. The plasmids, which contained the shRNA and the negative control were obtained from Gene Pharma (ShangHai, China). The negative control plasmid and the plasmid containing ALDOA-HA were purchased from OBiO Technology (Shanghai, China). We packaged these plasmids into virus particles using HEK 293 T cells and determined the viral titers. In order to establish stable KCNQ1OT1-knockdown cell lines or the ALDOA-overexpressing cell lines, the target cells were co-infected with 1 × 10^8^ lentivirus-transducing and polybrene (Sigma, MO, USA). Furthermore, 2.5 μg/mL puromycin or 10 mg/mL blasticidin were used to screen the infected cells after 72 h. The efficiency of the knockdown or overexpression was verified by western blot or qRT-PCR.

Control mimics, miR-34a-5p mimics, miR-34c-5p mimics, miR-449a mimics, miR-449b-5p mimics, miR-877-3p mimics, control inhibitor, and miR-34c-5p inhibitor were obtained from Gene-Pharma (Shanghai, China). The targeted miRNA mimic, miRNA inhibitor, and miR-control were transfected into U-2OS and 143B cells at a concentration of 50 nM by using Lipofectamine 3000 (Invitrogen). hFOB1.19 cells were cultured at 60% confluence and transfected with ALDOA-specific small interfering (si) RNA or a negative control siRNA (GenePharma, Shanghai, China) using Lipofectamine^®^ RNAiMAX (Thermo Fisher Scientific, Waltham, MA, USA). The efficiency of the knockdown or overexpression was verified by qRT-PCR or western blot.

### Protein extraction and western blotting

Total proteins from the cells were extracted by using RIPA buffer (Thermo Fisher Scientific, Rockford, lL, USA) containing protease inhibitors (Roche, Mannheim, Germany). The western blotting assay was performed as previously described^43^. In general, all of the cellular proteins were extracted and 15–20 μg of proteins were separated by 5–10% SDS-PAGE gels. Following transfer of the proteins onto a membrane, the membranes were incubated overnight at 4 °C with the primary antibodies, including the antibodies to ALDOA (ab169544; Abcam, Cambridge, UK), α-Tubulin (#2125; Cell Signaling Technology, MA, USA), cleaved Caspase-3 (ab2302; Abcam, Cambridge, UK), cleaved Caspase-7 (#8438; Cell Signaling Technology, MA, USA), cleaved Caspase-9 (#9509; Cell Signaling Technology, MA, USA), CDK4 (#12790, Cell Signaling Technology, MA, USA) and Cyclin D1 (ab226977; Abcam, Cambridge, UK). The next day, the membranes were incubated with secondary antibodies after being washed. Finally, the membranes were detected using an ECL substrate (Share-bio, Shanghai, China).

### IHC staining

The IHC assay was conducted with methods as previously described^43^. Ki67 was detected using the corresponding primary antibodies ki67 (GB13030; Servicebio, Wuhan, China) at 1:200 dilutions.

### Cell proliferation assays and cell apoptosis assay

Cell proliferation assays, including CCK-8 assay, colony formation assay, and cell apoptosis assay, were performed as previously described^43^. Three independent experiments were performed.

### EdU incorporation assay

The EdU incorporation assay was performed using a BeyoClick™ EdU Cell Proliferation Kit (C0075S, Beyotime Biotechnology, Shanghai, China) according to the instructions. Briefly, target OS cells were planted in the 12-well chambers (Ibidi, Germany), and 200 μl EdU (50 μM) was added to each well and incubated for 2 h. Thereafter, the cell was fixed with 4% paraformaldehyde for 15 min and permeabilized with 0.3% Triton X-100 for 10 min at room temperature. Next, the cells were washed again and labeled with 5 μg/ml of Hoechst 33342 for 30 min. Confocal microscopy (LSM 510, META laser scanning microscope, Zeiss) was used to acquire the images. Three independent experiments were performed.

### Cell cycle analysis

Cell cycle assay was performed using a Cell Cycle Analysis Kit (MultiSciences Biotech Co, Hangzhou, China) as previously described^43^. Three independent experiments were performed.

### Mouse xenograft assay

This experiment was performed with 6-week-old male BALB/C nude mice. Mice were manipulated and housed as previously described^41^. All animal studies were approved by the Research Ethics Committee of East China Normal University. Firstly, the mice were randomly divided into several groups and subcutaneously injected with 1.5 × 10^6^ of the target cells (*n* = 5 per group). Volume of the xenografted tumor was measured every 5 days and was calculated with the following formula: volume (mm^3^) = (length × width^2^)/2. On day 20, all mice were euthanized, and the tumors were collected and measured. Tissue samples were then fixed for further immunohistochemistry assay analysis.

### TUNEL assay

TUNEL assay was performed to quantify the proportion of apoptotic cells in the xenograft tumors as previously described by using a TUNEL kit (Roche, Basel, Switzerland)^43^.

### Measurement of cellular glycolysis and oxidative phosphorylation

Glycolysis was measured as the rate of extracellular acidification (ECAR), and oxidative phosphorylation was measured as the OCR via use of the XF96 metabolic flux analyzer (Seahorse Biosciences, Billerica, MA, USA) as previously described^43^. Three independent experiments were performed.

### Measurement of cellular ATP level and lactate production

An ATP assay kit (Promega, Madison, WI) was used to measure the cellular ATP level according to the manufacturer’s instructions. Bioluminescence was determined on a fluorescence luminometer (PerkinElmer, Waltham, MA). The ATP level was calculated from a standard curve. A lactate assay kit (BioVision, USA) was used to detect extracellular lactate levels according to the manufacturer’s protocol. All values were normalized to the cellular protein level. Three independent experiments were performed.

### Dual-luciferase reporter assay

The ALDOA 3ʹUTR sequence containing the predicted binding site of miR-34c-5p in ALDOA and the mutant sequence or the KCNQ1OT1 3ʹUTR sequence containing the predicted binding site of miR-34c-5p in KCNQ1OT1 and the mutant sequence were synthesized and sub-cloned into the pmirGLO Vector. U-2OS and 143B cells were co-transfected with the luciferase reporters, along with miR-34c-5p mimic, or the negative control using Lipofectamine 2000 (Invitrogen). Following 48 h, the relative luciferase activity was measured with the dual-luciferase reporter assay system (Promega, Madison, USA). Three independent experiments were performed.

### RIP assay

RIP was performed using a Magna RIP RNA-Binding Protein Immunoprecipitation Kit (Millipore, MA, USA) according to the manufacturer’s protocol. In brief, 1 × 10^7^ U-2OS and 143B cells transfected with pMS2bp-GFP and MS2, MS2-KCNQ1OT1, or miR-34c-5p mimic, and NC mimic were lysed in the RIP lysis buffer containing a protease inhibitor cocktail. Next, the cell supernatant was incubated with the RIP buffer containing a magnetic bead conjugated with antibodies against GFP (ab290, Abcam, UK), human AGO 2 (C34C6, CST, MA, USA) or the control normal mouse IgG (AP101, Millipore). Then the DNA and protein in the RIP complex were removed by using RNase-free DNase I and Proteinase K consecutively. Subsequently, the gained immunoprecipitated RNA was isolated and subjected to qRT-PCR to detect the enrichment of GFP, ALDOA, miR-34c-5p, or KCNQ1OT1. Three independent experiments were performed.

### RNA pull-down assay

The commercially synthesized biotin-labeled KCNQ1OT1 was purchased from Genepharma (Shanghai, China) and transfected into U-2OS and 143B cells for 48 h. Then, the cell lysate was incubated with Dynabeads M‐280 Streptavidin (Sigma, MO, USA) according to the manufacturer’s instructions. TRIzol was used to elute and purify the interacted RNA complex, and qRT-PCR was used to measure the expression level of miR-34c-5p. Three independent experiments were performed.

### Immunofluorescence (IF)

A microarray containing tissue from 40 OS patients was obtained from Alena Biotechnology Co., Ltd. (Xi’an, China). Immunofluorescence was performed as previously described^41^. Antibodies to ALDOA (CY7206; Abways, Shanghai, China) were used in IF. Confocal microscopy (LSM 510, META laser scanning microscope, Zeiss) was used to acquire the images. The intensities of ALDOA staining were scored using the following staining criteria 0–5% was scored as 0; 6–35% was scored as 1; 36–70% was scored as 2; and >70% was scored as 3. A total score <2 was considered to represent the negative expression, and a score ≥2 was defined as a positive expression. The scoring was performed blind and determined by two senior pathologists.

### Fluorescence in situ hybridization (FISH)

A microarray containing tissue from 40 OS patients was obtained from Alena Biotechnology Co., Ltd. (Xi’an, China). OS tissue sections were hybridized with the lncRNA KCNQ1OT1 (BioTNT Biotechnologies, Shanghai, China) and miR-34c-5p probes (Servicebio, Wuhan, China). Probe mix was denatured at 85 °C and hybridization was conducted at 65 °C overnight. Sections were washed using reducing concentrations of saline sodium citrate. Then slides were treated with 5% blocking solution for 30 min at room temperature. Each section was incubated with 100 μl HRP-labeled anti-DIG antibody at 1:500 in blocking buffer overnight at 4 °C. Then washed with TBS and TSA staining solution was created with a Perkin-Elmer TSA Plus kit according to the manufacturer’s instructions. Incubated in DAPI-containing TBS, then rinsed in water, air dried, and mounted in an aqueous fluorescence mounting media. Confocal microscopy (LSM 510, META laser scanning microscope, Zeiss) was used to acquire the images. The intensities of KCNQ1OT1 and miR-34c-5p staining were scored using the following staining criteria: 0–5% was scored as 0; 6–35% was scored as 1; 36–70% was scored as 2; and >70% was scored as 3. A total score <2 was considered to represent negative expression, and a score ≥2 was defined as a positive expression. The scoring was performed blind and determined by two senior pathologists.

### Database analysis

The data from the R2 database was used to analyze the relationship between ALDOA expression and the overall survival of the OS patient (https://hgserver1.amc.nl/cgi-bin/r2/main.cgi). The data from TargetScan (http://www.targetscan.org/), miRDB (http://www.mirdb.org/), and StarBase (http://starbase.sysu.edu.cn/index.php) were used to analyze potential interaction genes of miR-34c-5p or KCNQ1OT1.

### Statistical analyses

All statistics were performed using GraphPad Prism 7.0 and SPSS 16.0 software. Two-tailed Student’s *t*-test was used to calculate the statistical significance between groups. The chi-square test (SPSS 17.0; IBM, Armonk, NY, USA) was used to evaluate the correlation between the expression of KCNQ1OT1, miR-34c-5p, and ALDOA in human OS tissues. All data from the experiments were expressed as the mean ± standard deviation (SD). The statistical results obtained from the independent and randomized experiment, three replicates, and the *p-*value < 0.05 was considered statistically significant (*p* values > 0.05 = ns, *p* values < 0.05 = *, *p* values < 0.01 = **, *p* values < 0.001 = ***).

## Supplementary information

Supplementary figure 1

Supplementary figure 2

Supplementary figure 3

Supplementary figure 4

Supplementary figure 5

Supplementary figure 6

Supplementary figure legend

Supplementary table1
