# Supplementary figures and images for: LncRNA KCNQ1OT1 sponges miR-34c-5p to promote osteosarcoma growth via ALDOA enhanced aerobic glycolysis
Source: Cell Death Dis. 2020 Apr 24;11(4):278. doi: 10.1038/s41419-020-2485-1 (PMC7181648; doi:10.1038/s41419-020-2485-1)

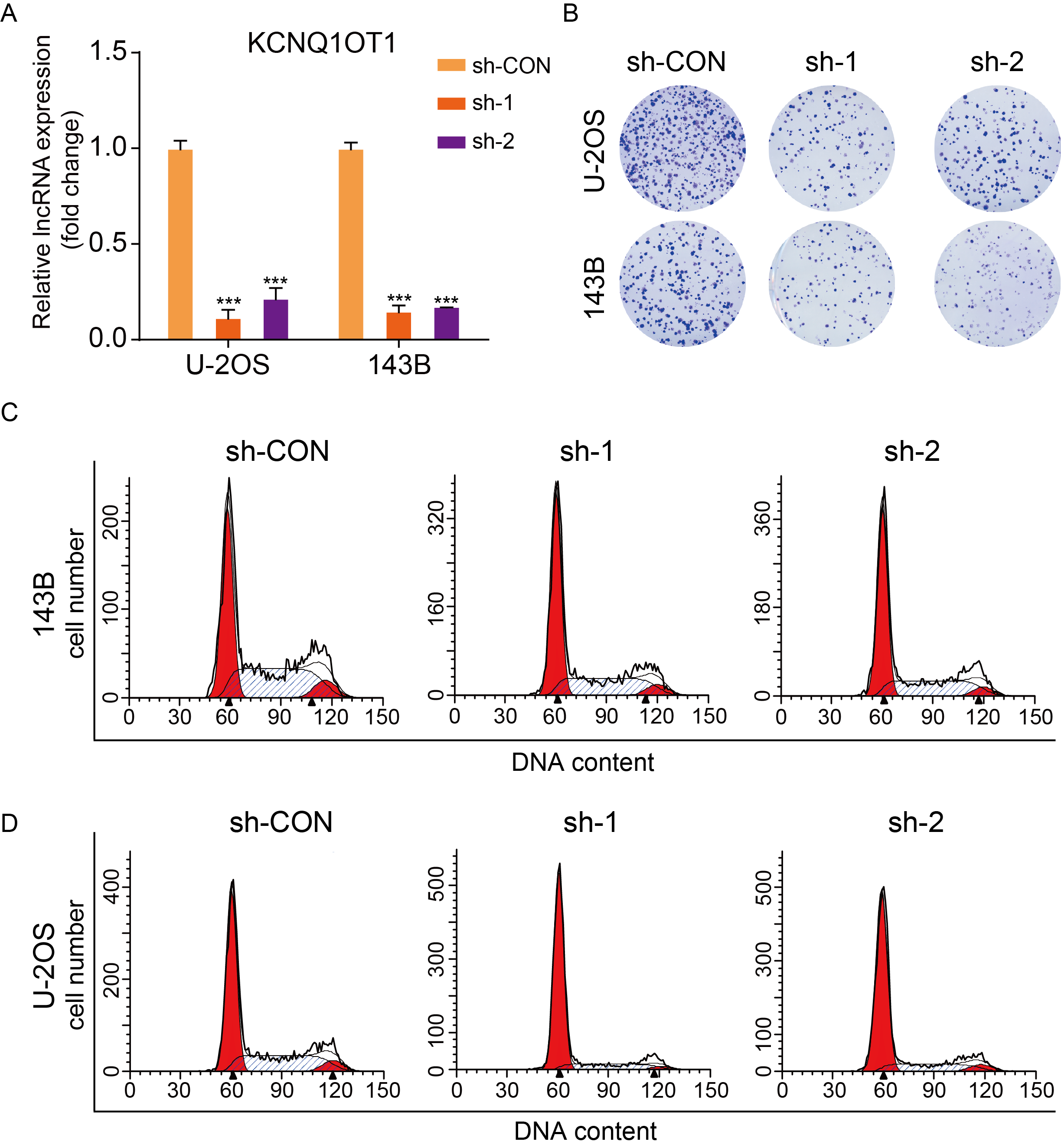

Supplement: Supplementary file 1 — Supplementary figure 1 [file 41419_2020_2485_MOESM1_ESM.png]

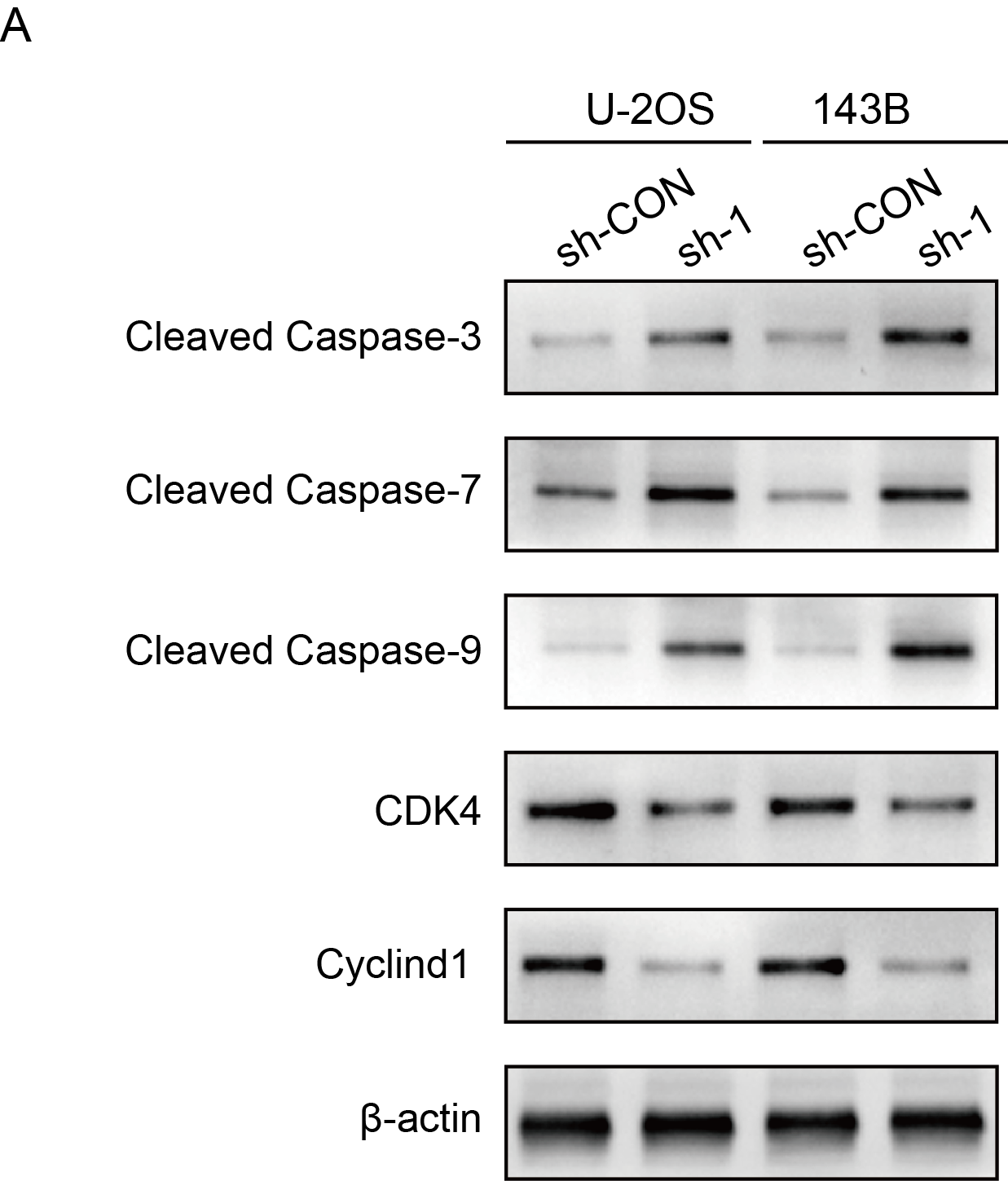

Supplement: Supplementary file 2 — Supplementary figure 2 [file 41419_2020_2485_MOESM2_ESM.png]

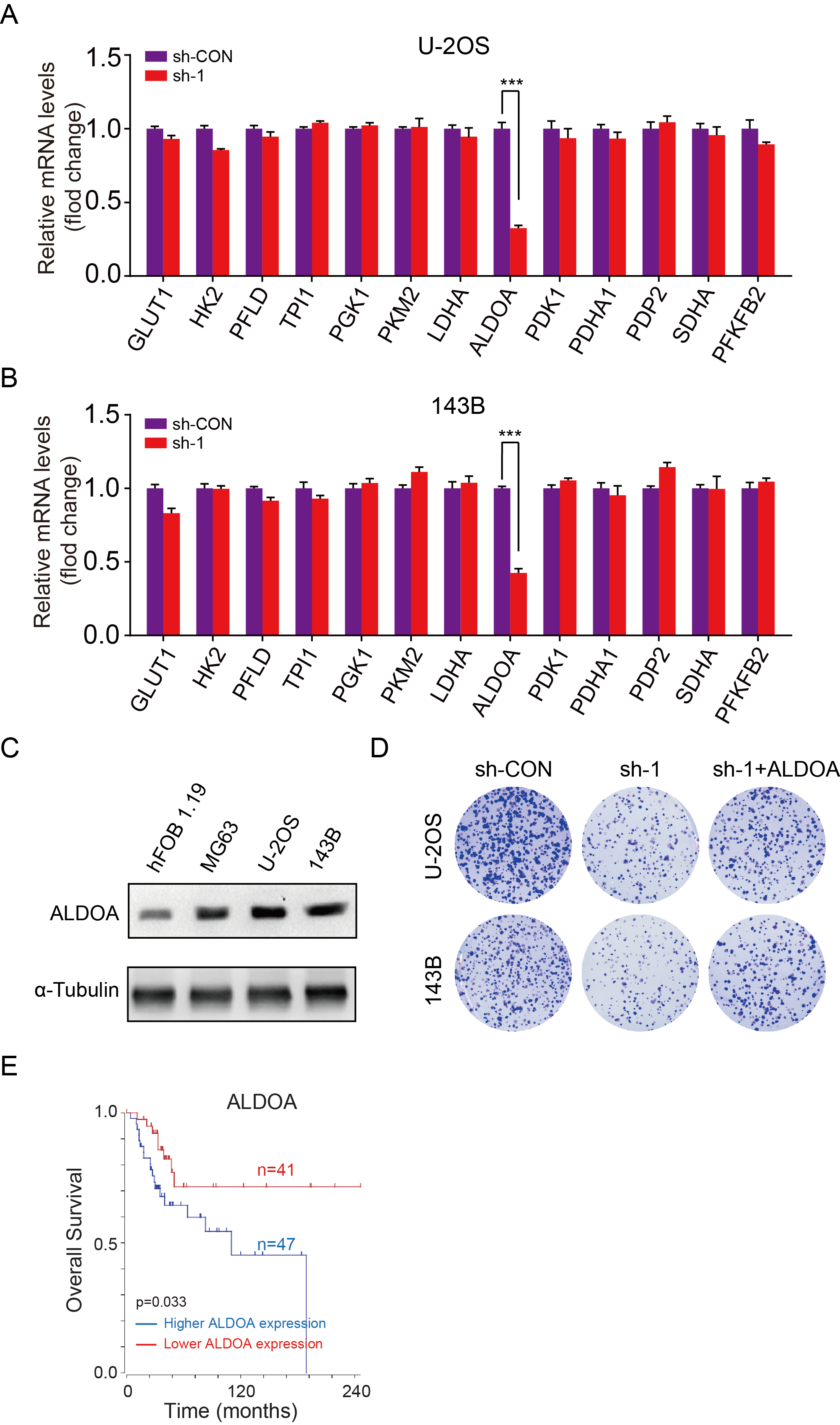

Supplement: Supplementary file 3 — Supplementary figure 3 [file 41419_2020_2485_MOESM3_ESM.png]

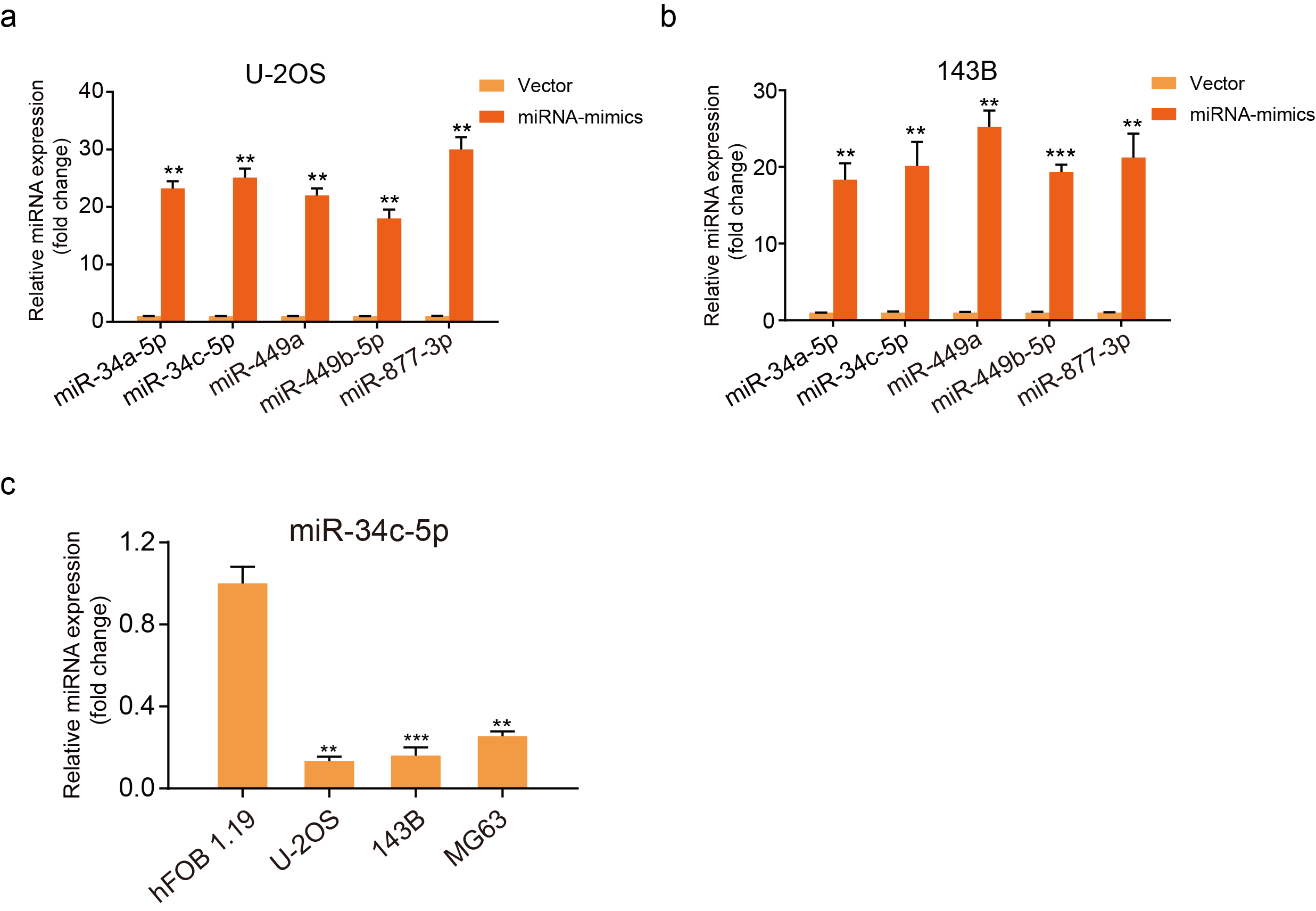

Supplement: Supplementary file 4 — Supplementary figure 4 [file 41419_2020_2485_MOESM4_ESM.png]

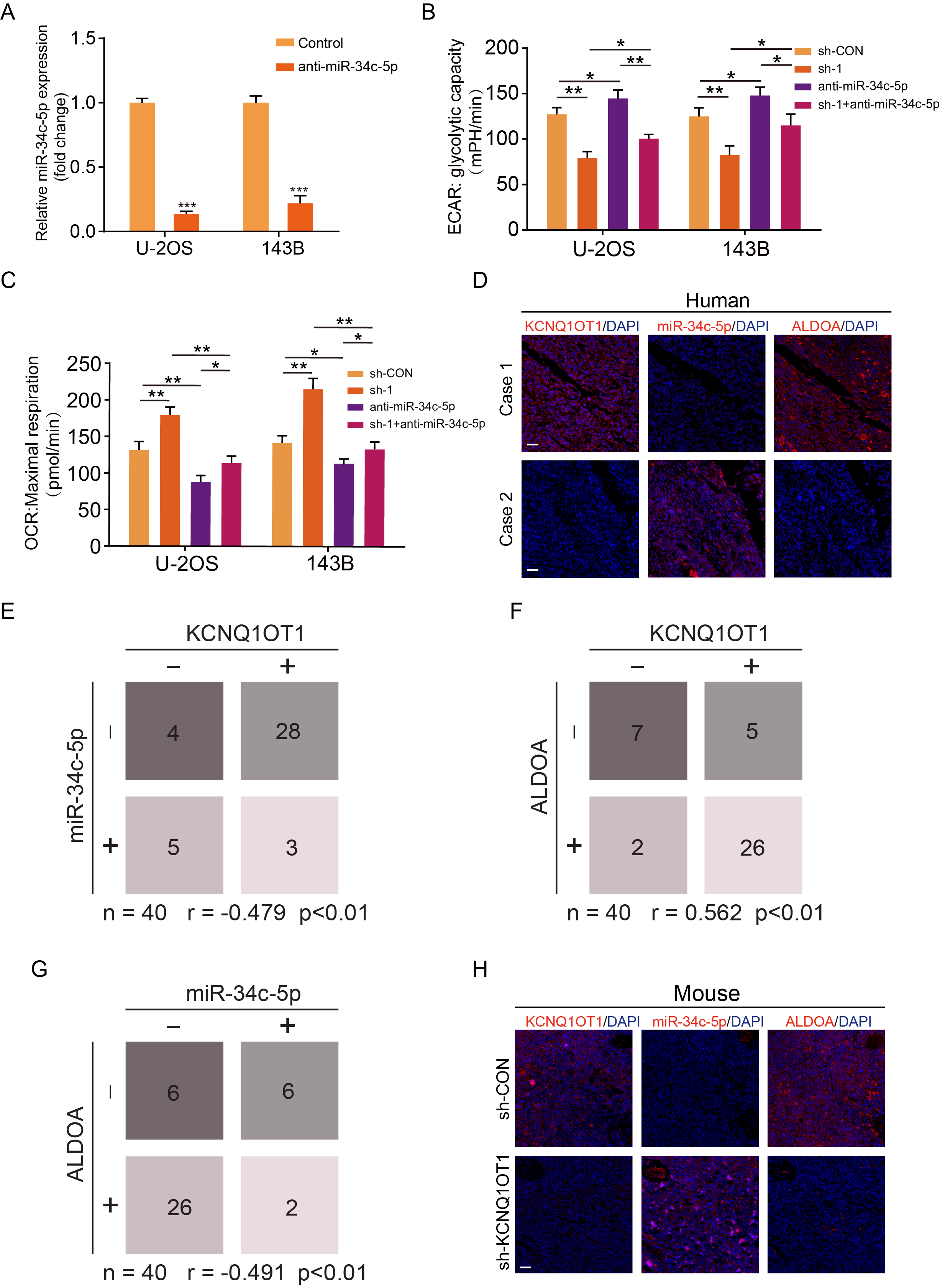

Supplement: Supplementary file 5 — Supplementary figure 5 [file 41419_2020_2485_MOESM5_ESM.png]

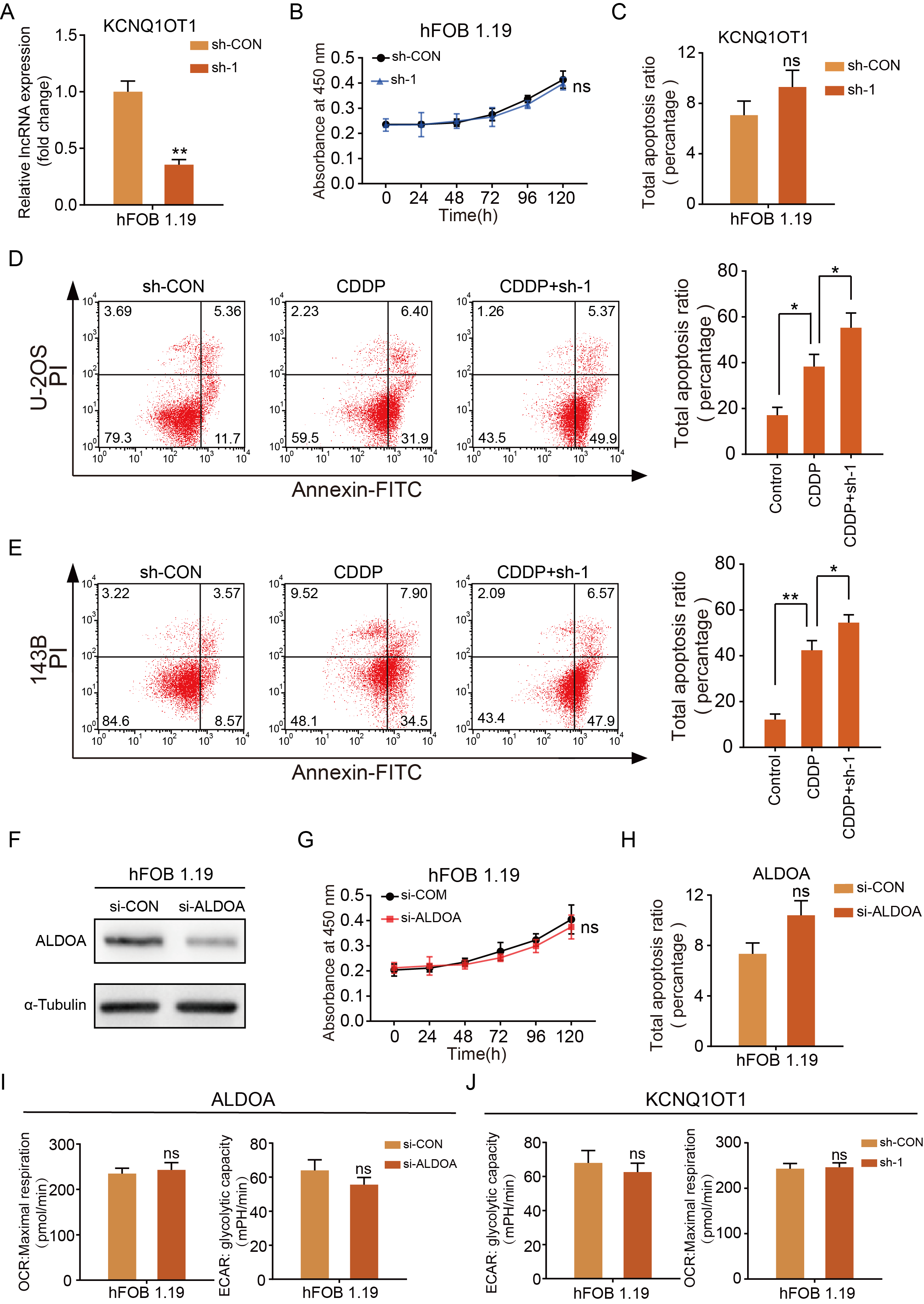

Supplement: Supplementary file 6 — Supplementary figure 6 [file 41419_2020_2485_MOESM6_ESM.png]
