# Supplementary material for: LncRNA KCNQ1OT1 sponges miR-34c-5p to promote osteosarcoma growth via ALDOA enhanced aerobic glycolysis
Source: Cell Death Dis. 2020 Apr 24;11(4):278. doi: 10.1038/s41419-020-2485-1 (PMC7181648; doi:10.1038/s41419-020-2485-1)
Supplement: Supplementary file 7 — Supplementary figure legend [file 41419_2020_2485_MOESM7_ESM.docx]

**Supplementary Fig 1**

a. Interference efficacy of sh-RNA targeting of KCNQ1OT1 in U-2OS and 143B cells was determined by qRT-PCR. Values are means ± SD, ***p < 0.001 (Student's t-test).

b. Representative images of OS colony formation assay in sh-Control and sh-KCNQ1OT1 groups are shown.

c and d. Representative photographs of the cell cycle assay in U-2OS and 143B cells transfected with KCNQ1OT1 shRNA or negative control shRNA.

**Supplementary Fig 2**

a. Western blotting analysis of the levels of cleaved caspases 3, cleaved caspases 7, cleaved caspases 9, CDK4 and Cyclin D1 in U-2OS and 143B cell lines. β-actin acts as an internal reference.

**Supplementary Fig 3**

a and b. Expression of the genes in the glucose metabolic pathway were determined by qRT–PCR in sh-Control and sh-KCNQ1OT1 groups. Values are means ± SD, *** p < 0.001 (Student's t-test).

c. Western blotting analysis of the levels of ALDOA in hFOB1.19, MG63, U-2OS and 143B cell lines. α-Tubulin acts as an internal reference.

d. Representative images of OS colony formation assay in sh-Control and sh-KCNQ1OT1 groups are shown.

e. Kaplan-Meier analysis of the correlations between ALDOA expression and overall survival rate of 88 OS patients based on an online database (https://hgserver1.amc.nl/cgi-bin/r2/main.cgi).

**Supplementary Fig 4**

a and b. Overexpression efficacy of miRNA mimics in U-2OS and 143B cells was determined by qRT-PCR. Values are means ± SD,**p < 0.01,*** p < 0.001 (Student's t-test).

c. qRT-PCR analysis of the levels of miR-34c-5p in hFOB1.19, MG63, U-2OS and 143B cell lines. Values are means ± SD,**p < 0.01,*** p < 0.001 (Student's t-test).

**Supplementary Fig 5**

a. Interference efficacy of inhibitor targeting of miR-34c-5p in U-2OS and 143B cells was determined by qRT–PCR, *** p < 0.001 (Student's t-test).

b. Quantification of glycolytic capacity from the fig. 6e. Values are means ± SD *p<0.05, **p < 0.01 (Student's t-test).

c. Quantification of maximal respiration from the fig. 6f. Values are means ± SD *p<0.05, **p < 0.01 (Student's t-test).

d. Representative photographs of the expression patterns of KCNQ1OT1, miR-34c-5p and ALDOA in huamn OS tissues by IF and FISH. Scale bars = 50 μm.

e. A negative correlation between the expression pattern of KCNQ1OT1 and miR-34c-5p (*n* = 40, *r* = -0.479, *p* < 0.01).

f. A positive correlation between the expression pattern of KCNQ1OT1 and ALDOA (*n* = 40, *r* = 0.562, *p* < 0.01).

g. A negative correlation between the expression pattern of miR-34c-5p and ALDOA (*n* = 40, *r* = -0.491, *p* < 0.01).

h. Representative photographs of the expression patterns of KCNQ1OT1, miR-34c-5p and ALDOA in tumor tissues from subcutaneous xenograft mouse model by IF and FISH. Scale bars = 50 μm.

**Supplementary Fig 6**

a. Interference efficacy of sh-RNA targeting of KCNQ1OT1 in hFOB1.19 cell line was determined by qRT-PCR. Values are means ± SD, **p < 0.01 (Student's t-test).

b. Knockdown of KCNQ1OT1 has no obvious effect on the proliferation capability of hFOB1.19 cells using the CCK-8 assay. Values are means ± SD, non sensual (ns) (Student's t-test).

c. Knockdown of KCNQ1OT1 has no obvious effect on the apoptosis of hFOB1.19 cell. Values are means ± SD, non sensual (ns) (Student's t-test).

d and e. Cell apoptosis assay using U-2OS and 143B cells treat with CDDP or combination of CDDP and sh-KCNQ1OT. Representative images of apoptosis assay were shown in the left panel. Values are means ± SD *p<0.05, **p < 0.01 (Student's t-test).

f. Interference efficacy of si-RNA targeting of ALDOA in hFOB1.19 cell line was determined by western blotting.

g. Knockdown of ALDOA has no obvious effect on the proliferation capability of hFOB1.19 cells using the CCK-8 assay. Values are means ± SD, non sensual (ns) (Student's t-test).

h. Knockdown of ALDOA has no obvious effect on the apoptosis of hFOB1.19 cell. Values are means ± SD, non sensual (ns) (Student's t-test).

i and j. Silencing ALDOA or KCNQ1OT1 had no obvious effect on the ECAR or OCR of hFOB1.19 cells. Values are means ± SD, non sensual (ns) (Student's t-test).
